# Risk and Protective Factors of Recurrence after Catheter Ablation for Atrial Fibrillation

**DOI:** 10.31083/j.rcm2503081

**Published:** 2024-03-01

**Authors:** Xinwei Guo, Jingbo Li

**Affiliations:** ^1^Shanghai Sixth People’s Hospital Affiliated to Shanghai Jiao Tong University School of Medicine, 200233 Shanghai, China

**Keywords:** atrial fibrillation, catheter ablation, atrial fibrillation recurrence

## Abstract

Atrial fibrillation (AF) is a common disease and is effectively managed through catheter ablation (CA). However, post-ablation AF recurrence can compromise patient outcomes, making the identification of associated risk factors crucially important. Factors influencing poor clinical outcomes include age, female sex, body mass index (BMI), non-paroxysmal AF, and comorbidities including diabetes mellitus (DM) and obstructive sleep apnea (OSA). Furthermore, the selected ablation strategy and employed technology are pivotal to long-term success in maintaining sinus rhythm control. The mechanisms of AF recurrence are complex and multifactorial; no single predictor is definitive. Thus, a personalized assessment of each patient should be tailored to the individual situation. A high risk of relapse does not preclude the option of ablation therapy, but rather underscores the necessity to address and manage underlying conditions contributing to AF pathogenesis, aiming to mitigate the risk of recurrence.

## 1. Introduction

Atrial fibrillation (AF) is the most common encountered arrhythmia in clinical practice. In the past three decades, the global prevalence of AF has grown sharply, reaching over 60 million cases, a rise influenced by extended life spans and changes in lifestyles [1, 2]. The mechanism of AF is not yet clear. However, it is recognized that a complex interplay between electrical and structural heart changes, such as ion channel alterations, atrial fibrosis, and enlargement, contribute significantly to both the initiation and perpetuation of AF [3]. Risk factors including advancing age, obesity, obstructive sleep apnea (OSA), heart failure (HF), and diabetes mellitus (DM) are thought to be involved in the development of AF. The presence of AF is associated with stroke, HF, decreased quality of life, and increased risk of mortality [4, 5].

AF recurrence is common. Catheter ablation is an established therapeutic option for various kinds of cardiac arrhythmia and is commonly used in patients with symptomatic AF refractory to antiarrhythmic drugs [6]. Compared to medical treatment, ablation surgery can significantly reduce the risk of death, stroke, and hospitalization [7]. Despite advances in ablation techniques and devices, post-ablation AF recurrence remains a significant challenge, and the exact mechanisms of AF recurrence have yet to be clearly defined. A common phenomenon observed in patients with recurring AF is the electrical reconnection of pulmonary veins (PVs), which is acknowledged as a key contributor to AF relapse. While in late recurrent arrhythmias, the lower number of PVs reconnections, increased incidence of extrapulmonary triggers, and structural changes could imply other mechanisms [8, 9]. Factors such as age, female gender, the specific type of AF, existing comorbidities, and the chosen ablation strategy and technology are recognized as influencing the risk of AF recurrence. To provide additional clinical insight into AF, and find ways to improve patients’ prognosis, here we will review recent relevant articles, and discuss the known factors associated with the post-ablation recurrence of AF.

## 2. Clinical Parameters

### 2.1 Age

AF is an age-related disease. Older populations are more likely to develop AF and have more advanced, established diseases, which are known to be AF risk factors. A study with 571 Chinese patients showed that in elderly AF patients, ablation could decrease all-cause and cardiovascular mortality [9]. Analysis of different age groups within the CABANA (Catheter Ablation versus Antiarrhythmic Drug Therapy for Atrial Fibrillation) trial indicates that while the efficacy of catheter ablation (CA) in preventing AF recurrence is fairly consistent across ages, there is a slight uptick in recurrence rates of AF/atrial tachycardia (AT) as age increases over a four-year period [10]. Notably, patients under 65 years derive greater benefits from ablation, with reduced incidences of stroke, bleeding, and overall mortality compared to those receiving antiarrhythmic drugs [11]. A retrospective study reported that the rate of major complications is lower in patients under 45, and the AF-free survival rate without antiarrhythmic drugs of those patients is greater after ablation [12]. Thus, younger patients may experience a greater benefit from ablation therapy with fewer recurrences and complications.

Older populations are more likely to experience additional risk factors related to recurrence, including DM, HF, and additional comorbidities. The risk factors for different aged populations vary. Uemura T *et al*. [13] reported that for younger paroxysmal AF patients (age <75 years), DM and female sex are related to atrial arrhythmia recurrence after CA, while for patients aged >75 years, only DM is associated. Furthermore, supraventricular ectopic complexes, known triggers of paroxysmal AF, are only associated with recurrent AF in older patients [14]. One single risk factor could not effectively predict AF recurrence.

### 2.2 Gender

Sex plays a significant role in the baseline clinical characteristics of patients and post-ablation outcomes. Several studies have shown that females undergoing ablation tend to be older, more symptomatic, and face a higher risk of complications compared to males [15, 16]. The mechanism behind these outcomes is not clear [15, 16]. Outcomes of ablation also vary from gender. The results from a retrospective study [17] showed that women who underwent ablation therapy experienced a higher proportion of paroxysmal AF and lower arrhythmic-free survival beyond one year following the procedure. A real-world study composed of 21 million U.S. patients showed that the hospitalization rate due to AF within one year was higher in females following ablation [18]. Furthermore, females were less likely to opt for repeat ablation following AF recurrence. However, the gender effect is not consistent among different types of AF. Female patients with persistent AF have a higher risk of AF/AT recurrence after radiofrequency CA [19]. While for paroxysmal AF patients, no significant difference in AF/AT-free survival time after ablation between males and females was observed [20].

The pathophysiological mechanisms of AF are different between sex. Sex-related structural differences, hormones, and electrical heterogeneity play a role in AF development [21]. Furthermore, left ventricular dysfunction, left atrial (LA) remodeling, atrial fibrosis, and autonomic nervous dysfunction are associated with in poor CA outcomes in women [22, 23]. Efforts have been made to find out the mechanism of gender effect in AF recurrence. Further research is needed to achieve a clearer understanding of sex-specific differences in AF mechanisms, incidence, and prognosis, to provide better AF management and achieve better clinical outcomes.

### 2.3 Obesity

Obesity is a well-established risk factor for AF recurrence after ablation. While the pathophysiological link between obesity and AF remains incompletely understood, there are associations between hemodynamic alterations and LA remodeling [24, 25]. Additionally, epicardia adipose tissue (EAT) contributes to the development of AF and is known to secrete cytokines with proinflammatory function and proliferative effects, increasing the risk of AF recurrence [26]. There is a strong correlation between body mass index (BMI) and EAT. BMI could be an independent predictor of AF relapse but is irrelevant to complications [27]. A meta-analysis with 26 studies demonstrated the relationship between BMI and AF recurrence after CA, and both obesity and being overweight (BMI >28 kg/$m^{2}$) were significantly associated with AF recurrence [28]. Furthermore, a BMI >28 kg/$m^{2}$ could be a predictor factor of recurrent AF, while weight loss could reverse the pathophysiological pathway of AF, hence decreasing the recurrent AF [28]. It has also been reported that weight loss was related to an increased duration of recurrent AF freedom, and risk factors coexisting with both obesity and atrial remodeling could be attenuated by weight loss [29]. Furthermore, results from a single-center retrospective study with 601 patients showed that pre-ablation weight loss was associated with longer freedom time from AF in overweight patients [30]. The benefit of pre- and post-ablation weight loss should be confirmed in further studies.

## 3. Results Comorbidities

### 3.1 Diabetes Mellitus

Patients with DM experience a higher risk of AF, worse AF symptoms, lower quality of life, and increased risk of death [31, 32, 33]. The link between DM on AF remains an issue of debate. As generally discussed, DM is associated with proarrhythmic electrophysiologic changes, atrial fibrosis, oxidative stress, and over-activated inflammation, contributing to the progress of atrial structural, electrical, and autonomic remodeling, which plays an important role in initiating and maintaining AF [34, 35].

Catheter ablation is safe in DM patients with comparable occurrence of complications [36]. Despite the comparable incidence of preprocedural complications among DM and non-DM patients, DM is associated with a higher rate of atrial arrhythmia recurrence, especially for patients with persistent AF [37, 38]. However, a meta-analysis [39] of 15 retrospective and randomized controlled trials (RCTs) reported that the overall complication of CA in patients with DM and the arrhythmia-free survival time after CA were both similar to that reported among the general populations. This study provided indirect evidence that the outcomes of CA in patients with DM are comparable with those in patients without DM, especially in young patients in younger patients with satisfactory glycemic control.

Lower basal glycated hemoglobin (HbA1c) is associated with longer maintenance of sinus rhythm after CA. Elevated glucose level in patients with diabetes could affect the biatrial substrate properties, leading to a higher recurrence rate after CA [40]. In a retrospective study with 351 patients, the preablation HbA1c levels of 47 patients with DM were recorded to assess their blood glucose levels [38]. Although there was a trend towards higher AF recurrence with increased preablation HbA1c levels, this data did not reach statistical significance [38]. This result might be related to the limited sample size within the study [38]. In a separate study [41] with 298 patients, the long-term AF recurrence was significantly lower in patients with preablation HbA1c levels <7%. After at least 12 months follow-up, the increase in HbA1c was related to higher AF recurrence, while >10% reduction in HbA1c lead to longer free from AF time [41]. Similarly, Lu *et al*. [42] found that a higher basal HbA1c level was accompanied by a lower arrhythmia-free survival, and HbA1c was an independent predictor of recurrent atrial tachyarrhythmia in patients with type 2 DM and paroxysmal AF (PAF).

While DM could increase the incidence of AF, it might not worsen the outcomes of CA for AF. The HbA1 level could predict the recurrence after CA, which could relate to atrial remodeling with worse blood glycemic control. However, further prospective studies with greater sample size need to be performed with the HbA1c level recorded during follow-up, to clarify the relationship between blood glycemic fluctuations and the recurrence of arrhythmia after AFCA. Sodium-glucose cotransporter 2 inhibitors (SGLT2i), when used as oral antidiabetic drugs, have been correlated with a lower rate of AF/AT recurrence following CA in AF patients with type 2 DM [43, 44]. This benefit appears to be separate from their blood sugar-lowering effects, suggesting that SGLT2i may influence atrial remodeling directly. The exact mechanisms of how SGLT2i contribute to these outcomes, however, warrant further investigation.

### 3.2 Obstructive Sleep Apnea

A diagnosis of OSA has been identified as a contributor to AF recurrence following CA [45, 46]. Commonly presenting alongside obesity, DM, and increased age, OSA diagnosis can be time-intensive and costly, leading to a substantial underdiagnosis in AF patients [47]. Consequently, there is a pressing need for an efficient and accessible method to identify those at higher risk. The STOP-BANG questionnaire (Snoring, Tiredness, Observed apnea and blood Pressure-Body mass index, Age, Neck circumference and Gender), as a simple and widely accepted screening tool for OSA, has been shown to independently predict AF recurrence in patients without previously diagnosed OSA [48]. This offers a potential and assessable method to evaluate patients’ OSA-related risks prior to CA [48]. A meta-analysis demonstrated the association between OSA and AF recurrence, and it also indicated that continuous positive airway pressure (CPAP) therapy could reduce the risk of AF recurrence [49]. Novel therapies targeted at the autonomic modulation involved in the pathogenesis of AF in OSA were established in several preclinical studies, and their safety and effectiveness should be confirmed in further clinical studies [50].

### 3.3 Heart Failure

A safe and effective method of rhythm control for AF patients with HF, CA has been associated with a reduced rate of mortality and HF-related hospitalizations compared to medical therapy, alongside a reduction in AF burden [51, 52, 53]. HF could be divided into HF with preserved ejection fraction (HFpEF) and HF with reduced ejection fraction (HFrEF) by the left ventricular ejection fraction (LVEF). According to Sohns *et al*. [54], the post-ablation improvement in LVEF was independent of the severity of left ventricular dysfunction, thus AF ablation should be performed during early stages. Most studies focused on the rate of mortality and set the incidence of death or hospitalization as the primary endpoint rather than AF recurrence. A study [55] focused on the long-term outcomes over a median 8-year follow-up in AF patients with HFrEF showed that mortality for patients with AF and HFrEF remained unacceptably high, while the mortality and long-term AF recurrence were similar in early routine CA and delayed selective CA with a high rate of repeat procedure and application of antiarrhythmic drugs However, this study further supported that CA was associated with a decreased incidence of all cause death compared with medical rate control [55]. For AF patients with HFpEF, the coexistence of AF and HFpEF could lead to a higher risk for AF recurrence following cryoballoon ablation (CBA) [56]. Neither relief of HF-related symptoms or quality of life improvements were seen in patients with HFpEF after pulmonary veins isolation (PVI). Usually, it is hard to diagnose AF with HFpEF due to similar symptoms and signs. The study cohort was limited in sample size, thus further studies are needed to validate these results. Wang *et al*. [57] found that Sacubitril/Valsartan could reduce AF recurrence after CA in patients with persistent AF. This improvement could be related to Sac/Val improve atrial remodeling [58].

## 4. Biomarkers and Physical Tests

### 4.1 Biomarkers

In addition to glycemic biomarkers like Hb1A1c that have predictive value for AF recurrence, natriuretic peptides, which reflect atrial remodeling, are also associated with recurrence rates. Higher baseline concentrations of N-terminal pro B-type natriuretic peptide (NT-proBNP) have been linked to CA failure [59]. A recent meta-analysis of 61 studies demonstrated that patients with AF recurrence have higher baseline levels of multiple peptides, indicating they could be used as recurrence predictors [60]. More specifically, these included atrial natriuretic peptide (ANP), B-type natriuretic peptide (BNP), NT-proBNP, and mid-regional pro A-type natriuretic peptide (MR-proANP) [60]. Furthermore, significantly higher baseline BNP levels were reported in women, influenced by other factors, resulting in a poorer predictive role of AF recurrence in females [61]. C-reactive protein (CRP) is related to inflammation reaction and baseline serum concentrations of high-sensitive CRP (hs-CRP) are associated with recurrence after CA [62, 63]. However, a study demonstrated that the post-ablation changes of hs-CRP rather than the baseline of hs-CRP were associated with poor CA outcomes [64]. Transforming growth factor (TGF)-β1 is related to the degree of atrial fibrosis and the serum concentration is associated with AF recurrence in non-PAF patients [65]. The post-ablation level of TGF-β1 should be further investigated to imply the relationship between TGF-β1 and AF recurrence. An observational study reported that the rate of post-ablation worsening renal function (WRF), defined as a decline of estimated glomerular filtration (eGFR) >30% after CA, was substantially more common among patients with recurrent AF [66]. Adding to this, a retrospective study demonstrated a positive correlation between serum uric acid: creatinine ratio (UCR) and recurrent AF, indicating UCR is a predictive factor for AF recurrence [67]. These findings emphasize the importance of renal health monitoring and uric acid levels in patients undergoing CA for AF, potentially aiding in the risk stratification and management of such individuals.

### 4.2 Echocardiograph

Left atrial size is related to AF recurrence, and echocardiography is a convenient way to assess the left atrial size. A meta-analysis of 22 studies showed larger left atrial diameter increased the risk of AF recurrence after single CA [68]. Research from a Chinese cohort suggested a U-shaped correlation between left atrium diameter (LAD) and AF recurrence, indicating that both a smaller LAD (≤3.0 cm) and a larger LAD (>4.6 cm) can be predictive of recurrence [69]. These findings hint at potential racial differences in cardiac structure and necessitate further investigation into why smaller LAD is associated with recurrence. Left atrium volume (LAV) is more accurate in assessing LA size. A meta-analysis of 21 studies [70] demonstrated that higher LAV is associated with post-ablation AF recurrence. Pongratz *et al*. [71] reported that for patients with persistent AF (PeAF), left atrial appendage (LAA) volume was a more reliable predictor of recurrence compared to LAV, with an LAA volume >9.75 mL being a strong predictor of arrhythmia recurrence. For patients with normal LAV (LAV index <34 mL/$m^{2}$), LA strain during the contraction phase could be predictive for the recurrence of atrial tachyarrhythmia [72].

## 5. Ablative Procedure

### 5.1 Early Recurrence

The first three months following CA are considered a blank period, and atrial tachycardia following this point defined as AF recurrence. Any arrhythmia occurrence during the blank period is considered to be early recurrence. While the relationship and potential mechanisms between early recurrence and late recurrence are under investigation, early recurrence is frequently associated with PV reconnection or insufficient ablation and may be predictive of long-term clinical outcomes [73, 74]. Improving the ablation procedure could reduce early recurrence. Additionally, early recurrence may be a predictor of late recurrence. Periprocedural short-term steroid therapy has been shown to reduce early recurrence after CA of atrial fibrillation, but this procedure is not effective in preventing late AF recurrence within one year [75]. This finding lends support to the hypothesis that inflammation plays a significant role in early recurrence. The implications of effectively preventing early recurrence on long-term arrhythmia-free survival warrant further exploration to better understand its potential benefits and to develop more targeted therapeutic strategies.

### 5.2 Ablation Strategy

The seminal work by Haïssaguerre *et al*. [76] identified firings from PVs as crucial triggers for AF, establishing PVI as a cornerstone of AF ablation therapy. For patients with paroxysmal atrial fibrillation, PVI alone is generally considered an effective method to prevent AF recurrence with success rates of a single procedure approaching 80% [77]. However, non-PV triggers take an important role in the initiation and maintenance of non-PAF. The common sites are mitral regions, the interatrial septum, the left atrial posterior wall, the LAA, and other thoracic veins such as the superior vena cava, the coronary sinus, and the ligament of Marshall [78]. Different ablation strategies have been developed to improve lesion quality and durability with an acceptable safety profile in non-PAF patients. Some RCTs reported that PVI with additional linear lesions or substrate modifications was more effective than PVI alone in patients with non-PAF [79, 80]. Compared with CA alone, CA with Marshall ethanol infusion improved the sinus rhythm maintained in patients with PeAF [81]. Table 1 (Ref. [73, 82, 83, 84, 85, 86, 87, 88, 89, 90, 91]) summarizes recent clinical trials comparing the effectiveness and safety of different ablation strategies in patients with PeAF. The success rate of PVI alone in PeAF patients ranged from 40% to 70%, which was lower than that in PAF patients, and those results suggested extra ablation of non-PV area is necessary to achieve better long-term outcomes. However, the effectiveness of one approach for ablation in non-PAF is still an issue to debate, and no solid evidence shows one ablation strategy is superior. However, high voltage mapping after PVI could help distinguish patients who have a higher risk of recurrent AF, and ablation guided by low-voltage area ablation seems to achieve better outcomes [82, 92]. PVI alone is effective in PAF patients, while the best ablation strategy for PeAF remains to be determined by larger, multi-center randomized controlled trials. However, identification and ablation of abnormal LA substrate in individual patients could achieve better single-procedure CA outcomes.

**Table 1.** **Studies of different strategies for persistent AF**.

Study (year)	Strategy	Follow-up (month)	Results
Verma *et al*. (2015) [83]	PVI alone vs. PVI + CFAE vs. PVI + linear ablation (LA roof and mitral valve isthmus)	18	Similar freedom of recurrent AF/AT (49% in PVI + CFAE group vs. 46% in PVI + linear ablation group vs. 59% in PVI alone group); complications: 1 cardiac tamponade in PVI alone group, 1 pericarditis and 2 TIA/stroke in PVI + CFAE, 2 pericarditis, 2 cardiac tamponade and 1 TIA/stroke in PVI + linear ablation group).
Bai *et al*. (2016) [84]	PVI alone vs. PVI + PWI	38	↑free from AF/AT recurrence survival (40% in PVI + PWI group vs. 10% in the control group, Log-rank *p* < 0.01).
Fink *et al*. (2017) [85]	PVI + substrate modification vs. PVI alone	12	↑Ablation time, procedure duration, fluoroscopy time and radiation dose; similar freedom from atrial tachyarrhythmia; complications: cardia tamponade in 4%, groin bleeding requiring transfusion or surgical therapy in 7%.
Yorgun *et al*. (2017) [73]	PVI alone vs. PVI + LAAI	12	↑ Total procedure time and fluoroscopy time; ↑AF/AT free survival (86% in PVI + LAAI group vs. 67% in the control group), no complications observed.
Lee *et al*. (2019) [86]	PVI alone vs. PVI + PWI	16.2 ± 8.8	↑ Procedure time, ablation time; similar fluoroscopy time; complications (5.9% in PWBI group vs. 6.6% in control group); recurrent AF/AT 26.5% in PVI + PWI group vs. 23.8% in control group.
Inoue *et al*. (2021) [87]	PVI alone vs. PVI + CFAE and/or PWI	12	↑ Procedure time, energy delivery, fluoroscopy time; similar freedom from AF/AT (78.3% in the PVI-plus group versus 71.3% in the control group); complication rates were 2.0% in the PVI-alone group and 3.6% in the PVI-plus group.
Aryana *et al*. (2021) [88]	PVI alone vs. PVI + PWI	12	↑ Left atrial dwell time and total procedure time; ↓intraprocedural cardioversions; ↓left atrium diameter within 6 months after ablation; ↓incidence of recurrent atrial fibrillation (25.5% vs. 45.5%; *p* = 0.028).
Yang *et al*. [82]	PVI alone vs. PVI + CFAE and/or linear ablation	18	AF/AT-free survival had no significant difference between PVI + extra ablation group and PVI alone group (67.2% vs. 67.4%); the success rate was higher in patients with normal LA substrate comparing with that in patients with low-voltage area (84.8% vs. 60.9%).
Kistler *et al*. [89]	PVI with PWI versus PVI alone	12	Rates of freedom from AF/AT were similar (52.4% in PVI + PWI group vs. 53.6% in PVI group).
Masuda *et al*. [90]	PVI-alone vs. PVI + linear ablation or CFAE	36	↓Recurrent AF/AT (26.9% in PVI + linear ablation or CFAE group versus 37.5% in the control group); the effectiveness of PVI + extra ablation was only higher than PVI alone among patients >65 years old.
Yorgun *et al*. [91]	PVI alone vs. PVI + LAAI	30	↑ Procedure time and fluoroscopy time; ↑Freedom of recurrent AF/AT (75.7% in PVI + LAAI vs. 61.6% in the PVI alone group); ↓rate of early recurrent AF/AT (9.0% in PVI + LAAI group vs. 24.6% in control group).

PVI, pulmonary vein isolation; CFAE, complex fractionated atrial electrogram; AF, atrial fibrillation; AT atrial tachycardia; PWI, posterior wall isolation; LA, left atrial; LAAI, left atrial appendage isolation; TIA, transient ischemia attack.

### 5.3 Ablation Techniques

Radiofrequency and cryoablation are currently the predominant methods for creating ablation lesions. Many prospective RCTs have shown similar safety and efficiency for AF patients, and there is no solid evidence that one ablative technique is superior [93, 94, 95, 96]. To achieve better outcomes and fewer complications, some novel ablative techniques emerged. Compared with the standard ablation strategy, high-power and short-duration (HPSD) radiofrequency ablation has been shown to potentially reduce procedure time without increasing complication rates, and one RCT reported it may improve freedom from AF [97, 98, 99]. Pulsed field ablation (PFA) is a tissue-selective modality which could safely achieve durable PVI and left atrial posterior wall ablation in patients with PeAF [100]. A pooled analysis of three separate studies demonstrated that PVI performed with a PFA catheter is durable and safe with a low arrhythmia recurrence rate at one year [101]. A real-world study of European patients also supports the high efficacy and safety profile of PFA in AF treatment [102]. Furthermore, a multicenter RCT assessing PVI using PFA, in comparison to cryoballoon or radiofrequency ablation, indicated that PFA was non-inferior to these established thermal ablation methods regarding both efficacy and safety [103].

Visually guided laser balloon ablation could be a reliable method to achieve persistent PVI [104]. A meta-analysis of 17 studies with 1188 patients demonstrated that the rate of 12-month freedom of atrial arrhythmia could reach 74.3% [105]. Compared with CBA and radiofrequency ablation, laser balloon ablation could decrease the rate of acute PVI failure and AF recurrence [106, 107].

### 5.4 Lesion Size

Recent observational studies and RCTs have not found improvements in the use of contact force (CF) -sensing ablation [108]. The ablation index (AI) is a novel ablation lesion marker that includes CF, ablation duration, and radiofrequency power. It was reported that the lower minimum AI was associated with PV reconnection which is relevant to AF recurrence [109]. Recently, a meta-analysis including 11 non-randomized studies of 2306 patients reported a significantly lower rate of atrial arrhythmia recurrence after ablation, with comparable safety to non-AI CA [110]. Given the relatively small size of cases and moderate quality of evidence, a large RCT is required to confirm these potential benefits of AI-guided CA.

### 5.5 Anesthesia Management

In 2011, an RCT reported the use of general anesthesia during the ablative procedure was associated with fewer PV reconnections and greater freedom from AF [111]. From 2010 to 2019, the number of AF ablation procedures increased, and the proportions of general anesthesia and deep sedation use increased [112]. It was reported that general anesthesia could reduce pain, improve catheter stability, increase contact force, and prevent PV reconnection, hence reducing AF recurrence after CA [113, 114]. It is possible that deep sedation could further reduce the incidence of PV activity and dormant PV conduction, not able to improve the freedom from AF recurrence [115]. Deep sedation and general anesthesia could provide sufficient control of movement, regular respiration, and increased mapping accuracy, causing better ablation lesion quality.

## 6. Conclusions

**Fig. 1.**
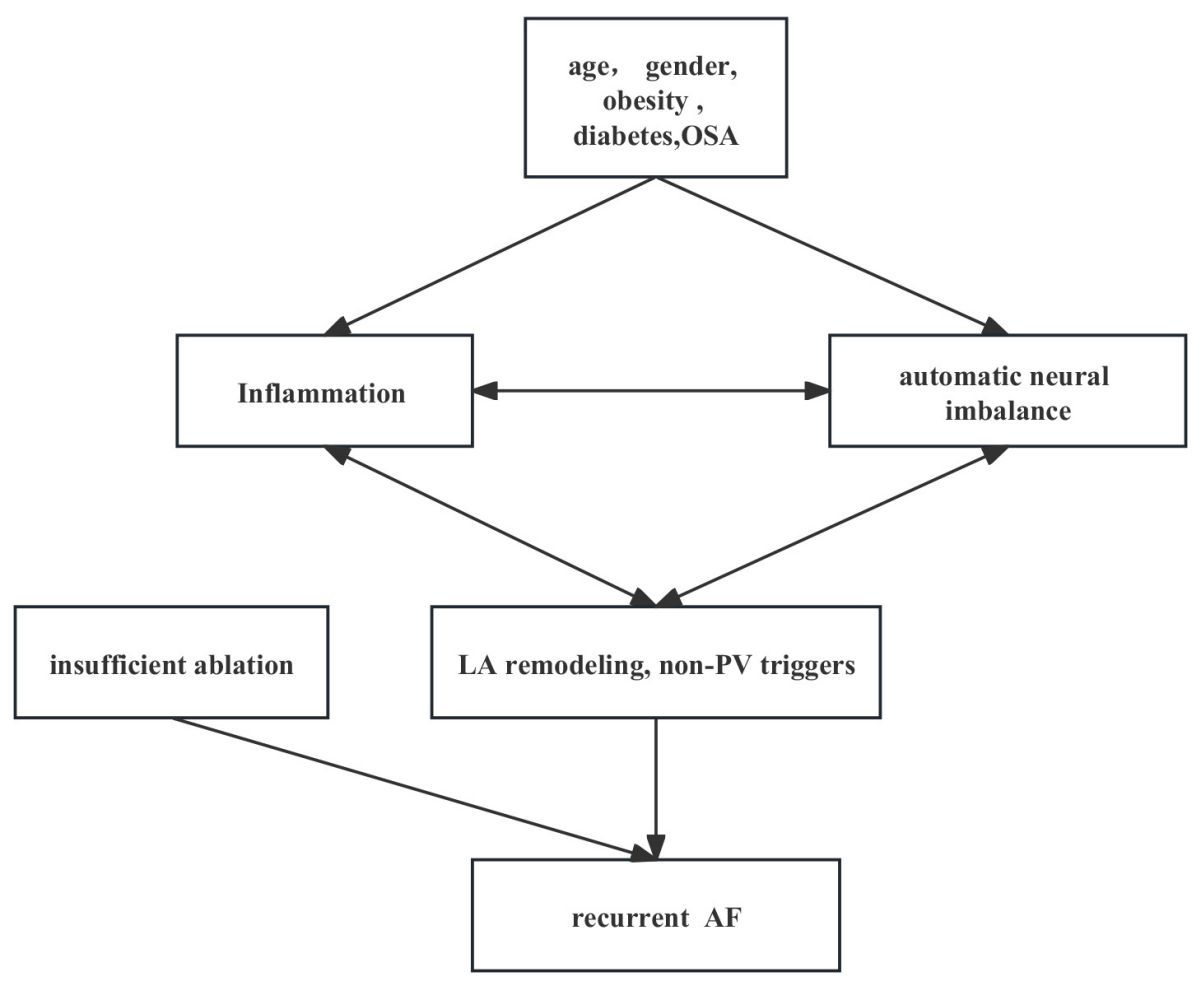
**Risk factors of AF recurrence**. OSA, obstructive sleep apnea; LA, left atrial; PV, pulmonary vein; AF, atrial fibrillation.

Recurrences of AF are common after CA and identifying risk factors for AF recurrence is of great importance. Fig. 1 shows the risk factors and potential mechanisms of AF recurrence. Mechanisms under recurrent AF are complex and undetermined, mainly involving inflammation, automatic neural atrial fibrosis, and remodeling. Age, female gender, BMI, non-paroxysmal AF, and coexistence comorbidities including DM and OSA are relevant. However, risk factors usually coexist with others and vary in different subgroups of patients, thus there is no single factor that could predict AF recurrence. Insufficient ablation and non-PV triggers could also contribute to recurrent AF. Controlling comorbidities and suitable ablation strategy and technology could increase long-term success in maintaining sinus rhythm. Each patient should be evaluated personally based on their situation and preferences. A high risk of relapse is not a contraindication for ablation therapy, and it is important to identify and intervene in disease or health conditions related to atrial fibrillation pathogenesis to reduce the risk of recurrent AF.
